# The anesthetic propofol shifts the frequency of maximum spectral power in EEG during general anesthesia: analytical insights from a linear model

**DOI:** 10.3389/fncom.2013.00002

**Published:** 2013-02-05

**Authors:** Axel Hutt

**Affiliations:** INRIA CR Nancy - Grand Est, Team CORTEXVillers-les-Nancy, France

**Keywords:** general anesthesia, propofol, neural fields, power spectrum, EEG

## Abstract

The work introduces a linear neural population model that allows to derive analytically the power spectrum subjected to the concentration of the anesthetic propofol. The analytical study of the power spectrum of the systems activity gives conditions on how the frequency of maximum power in experimental electroencephalographic (EEG) changes dependent on the propofol concentration. In this context, we explain the anesthetic-induced power increase in neural activity by an oscillatory instability and derive conditions under which the power peak shifts to larger frequencies as observed experimentally in EEG. Moreover the work predicts that the power increase only occurs while the frequency of maximum power increases. Numerically simulations of the systems activity complement the analytical results.

## 1. Introduction

General anesthesia (GA) is an important medical application in today's hospital surgery. Although GA is omnipresent in recent medicine, its underlying neural interactions have been a long-standing mystery. In the last decades, the anesthetic phenomena have attracted theoreticiens, e.g., (Steyn-Ross et al., 1999; Bojak and Liley, 2005; Hutt, 2011), who aim to describe mathematically some major experimental phenomena by population models (Steyn-Ross et al., 2004, 2012; Bojak and Liley, 2005; Hutt and Longtin, 2009; Hindriks and van Putten, 2012), or spiking-neuron models (McCarthy et al., 2008; Ching et al., 2010). Most theoretical studies aim to explain signal features of electroencephalographic (EEG) data observed during anesthesia. Such features comprise the diminution of α-activity accompanied by a subsequent enhancement of δ-activity while increasing anesthetic concentration (Gugino et al., 2001; Cimenser et al., 2011; Murphy et al., 2011) and the power enhancement of activity induced by some anesthetics (McCarthy et al., 2008; Ching et al., 2010). Another example is the increase of the frequency of maximum EEG-power to higher values as observed experimentally in several studies (Gugino et al., 2001; Ching et al., 2010; Murphy et al., 2011; Boly et al., 2012; Hindriks and van Putten, 2012). The current work focusses on the power enhancement and the frequency shift of maximum power while increasing the anesthetic concentration and gives insights into its origin by the analytical treatment of a linear neural field model.

One of the major objectives of this work is to answer the question whether it is possible to explain spectral EEG-features observed during GA by a low-dimensional linear model. The advantage of such a reduced model is the analytical tractability and an identification of underlying neural interactions or even the origin of the spectral feature. Here the difficulty is to find a simple model, that, however, still involves important, i.e., realistic and neural interactions. We are convinced that such a model has been found in a previous work (Hutt and Longtin, 2009). The present work will simplify further this spatio-temporal model while taking into account the biophysical effects of the anesthetic propofol on synaptic receptors and hence retaining the neurobiological plausibility.

The simplicity of the model will allow to reveal the effect of different actions of the anesthetic propofol on synaptic receptors on the frequency of maximum spectral power. Moreover, the work gives criteria under which conditions the frequency of maximum power increases with increasing propofol concentration and when it may decreases. In the analytical treatment, we will see that the power enhancement for larger propofol concentrations may be explained by an oscillatory instability and we predict that it always occurs while the frequency of maximum power increases.

## 2. Methods

### 2.1. The model

The neural field model under study (Hutt and Longtin, 2009) describes the evolution of the mean membrane potential of a neural population in a small spatial patch at spatial location *x* and at time *t*. Similar models have been derived and studied before (Wilson and Cowan, 1972; Amari, 1977; Ermentrout, 1998) and applied successfully to explain spatio-temporal neural activity observed experimentally (Ermentrout and Cowan, 1979; Huang et al., 2004; Angelucci and Bressloff, 2006; Schwabe et al., 2006). The population includes both excitatory and inhibitory neurons and takes into account excitatory and inhibitory synapses. Assuming that excitatory and inhibitory neurons exhibit identical effective membrane potentials, i.e., an identical difference between excitatory and inhibitory post-synaptic potentials, the mean excitatory and inhibitory post-synaptic potentials *V*_*e*_(*x*, *t*) and *V*_*i*_(*x*, *t*), respectively, obey

(1) $$\begin{array}{l} {\hat{L}}_{e}V_{e}(x,t)=a_{e}\int_{D}K_{e}(x-y)S_{e}[V_{e}(y,t)-V_{i}(y,t)]dy+I(x,t)\\ {\hat{L}}_{i}V_{i}(x,t)=a_{i}\int_{D}K_{i}(x-y)S_{i}[V_{e}(y,t)-V_{i}(y,t)]dy \end{array}$$

with the circular spatial population domain D of length *L*, i.e., assuming periodic boundary conditions. The model under study differs from some other previous models, e.g., by Liley and Bojak (2005), by the implementation of synaptic action, generation of action potentials or axonal connectivity [see also the work of Coombes et al. (2007) for a comparison of the current model and other models]. The functionals *S*_*e*_[·] and *S*_*i*_[·] are continuously increasing and represent the population firing rate of excitatory and inhibitory neurons, respectively. In the population the single neurons are connected by a complex system of axons from neuron somata to synapses. The kernels *K*_*e*_(*x*) and *K*_*i*_(*x*) are the probability density of such connections in the population. Here axonal transmission delay is neglected for simplicity although it is straightforward to include it in this type of model (Hutt and Longtin, 2009). In addition, the model considers excitatory and inhibitory synapses, ${\hat{L}}_{e}={\hat{L}}_{e}(d/dt)$ and ${\hat{L}}_{i}={\hat{L}}_{i}(d/dt)$ denote functional operators describing the corresponding temporal synaptic response phase and the factors *a*_*e*_, *a*_*i*_ represent the corresponding synaptic efficacies.

Mathematically, the differential operators are the inverse of the integral operators in

(2) $$V(t)=\int_{-\infty }^{t}h(t-\tau )P(\tau )\;d\tau$$

where *h*(*t*) is the synaptic response function, or more precisely the electric current response in the synaptic receptor to an impact of binding neurotransmitters (Koch, 1999). The function *P*(τ) > 0 is the mean pulse activity arriving at the synapses. In a reasonable approximation, the response function reads

$$h(t)=\frac{a}{\tau }e^{-t/\tau }$$

with the decay time constant τ and the synaptic efficacy *a* > 0. Then the response amplitude is *h*(0) = *a*/τ and the charge transferred in the receptor ρ = *a* is the time integral over the current flow. The corresponding differential operator stipulates $\hat{L}V(t)=aP(t)$ leading to

$$\hat{L}\left(\frac{\partial }{\partial t}\right)=\tau \frac{\partial }{\partial t}+1$$

and Equation (2) re-casts to

$$\tau \frac{\partial V(t)}{\partial t}+V(t)=aP(t).$$

These expressions hold for excitatory and inhibitory synapses.

The synaptic receptors are major targets of anesthetic agents. The present work considers the action of propofol on inhibitory synaptic and extra-synaptic GABA_*A*_-receptors. The former receptor is supposed to be a major anesthetic target (Franks and Lieb, 1994) and there is growing evidence that extra-synaptic inhibitory receptors may play an important role in anesthesia as well (Orser, 2006; Hutt, 2012). The subsequent sections consider effects on synaptic receptors due to the well-established experimental evidence. Hence, the synaptic parameters of inhibitory synaptic receptors depend on the anesthetic concentration and are parameterized by the factor *p* ≥ 1 (Steyn-Ross et al., 2001), i.e., the decay time of inhibitory synapses τ_2_ = τ_2_(*p*) and the corresponding synaptic efficacy *a*_*i*_ = *a*_*i*_(*p*) depend on *p*.

The input in Equation (1) fluctuates randomly in space and time with ξ(*x*, *t*) about a constant value *I*_0_ = const, i.e., *I*(*x*, *t*) = *I*_0_ + ξ(*x*, *t*). The random fluctuations are independent in space and time and thus obey 〈ξ(*x*, *t*)〉 = 0, 〈ξ(*x*, *t*)ξ(*y*, *T*)〉 = 2*D*δ(*t* − *T*)δ(*x* − *y*), where 〈·〉 denotes the ensemble average.

Considering the latter definitions of synaptic properties, anesthetic action and external input, the final model equations read

(3) $$\begin{array}{l} \;\tau_{1}\frac{\partial V_{e}(x,t)}{\partial t}=-V_{e}(x,t)+a_{e}\int_{D}K_{e}(x-y)S_{e}[V_{e}(y,t)-V_{i}(y,t)]dy+I_{0}+\xi (x,t)\\ \tau_{2}(p)\frac{\partial V_{i}(x,t)}{\partial t}=-V_{i}(x,t)+a_{i}(p)\int_{D}K_{i}(x-y)S_{i}[V_{e}(y,t)-V_{i}(y,t)]dy \end{array}$$

with the decay time of excitatory synapses τ_1_.

Assuming that the random fluctuations are small and do not affect the stationary state [in contrast to recent results gained from non-linear systems (Hutt et al., 2007; Hutt, 2008)], the stationary state *V*_*e*_(*x*, *t*) = *V*^0^_*e*_ = const, *V*_*i*_(*x*, *t*) = *V*^0^_*i*_ = const obeys *V*^0^_*e*_ = *a*_*e*_*S*_*e*_[*V*_−_] + *I*_0_, *V*^0^_*i*_ = *a*_*i*_(*p*)*S*_*i*_[*V*_−_] with *V*_−_ = *V*^0^_*e*_ − *V*^0^_*i*_ = *a*_*e*_*S*_*e*_[*V*_−_] − *a*_*i*_(*p*)*S*_*i*_[*V*_−_] + *I*_0_ (Hutt and Longtin, 2009).

### 2.2. Theoretical power spectrum

To compute the power spectrum, we employ the method of Greens function. Let us assume the activity variable vector **x**(*t*) ∈ ℛ^*N*^, the matrix **A**, the external input vector ξ(*t*) ∈ ℛ^*N*^, the Greens function matrix **G**(*t*) ∈ ℛ^*N* × *N*^ and

$$\dot{x}(t)=Ax+\xi (t).$$

Then, for *t* → ∞, the solution of the system is

(4) $$x(t)=\int_{-\infty }^{\infty }G(t-\tau )\xi (\tau )d\tau .$$

and the Greens function obeys

$$\dot{G}-AG(t)=1\delta (t)$$

with te unitary matrix **1** ∈ ℛ^*N* × *N*^. Applying the Fourier transform

(5) $$G(t)=\frac{1}{\sqrt{2\pi }}\int_{-\infty }^{\infty }\tilde{G}(\omega )e^{i\omega t}d\omega .$$

yields

(6) $$\tilde{G}(\omega )=\frac{1}{\sqrt{2\pi }}{\left(i\omega 1-A\right)}^{-1}$$

(7) $$=\frac{1}{\sqrt{2\pi }}\frac{F(i\omega )}{P(i\omega )}$$

with the matrix **F**(*i*ω) and the characteristic polynom *P*(*i*ω). The matrix **F** in Equation (7) includes the matrix elements of the inverse of *i*ω**1** − **A** and the characteristic polynom *P* represents the corresponding matrix determinant.

Inserting Equation (6) into (5) allows to compute **G**(*t*) by the residue theorem in functional analysis

(8) $$G(t)=2\pi i\sum_{n=1}^{r}Res(z_{n},t)\Theta (t)$$

with the Heaviside function Θ(*t*) and the residues matrix **Res**(*z*_n_, *t*) of **F**(*z*)/*P*(*z*) at the roots *z*_*n*_ of the characteristic equation *P*(*z*) = 0. The condition *t* > 0 considered by the Heaviside function is the mathematical condition for the validity of Equation (8) while it also guarantees the causality of the system response. Equation (8) together with Equation (4) determines the time dependence of the solution *x* and is computed explicitly in section 3.4.

Finally, the power spectral density matrix **S**(ω) of **x** is the Fourier transform of the auto-correlation function matrix 〈**x**^*t*^(*t*)**x**(*t* − *T*)〉 (Wiener-Khinchine Theorem) leading to

$$S(\omega )=2D\sqrt{2\pi }\tilde{G}(\omega ){\tilde{G}}^{t}(-\omega ),$$

where the high index *t* denotes the transposed vector or matrix.

## 3. Results

### 3.1. Effect of propofol

The effect of the anesthetic propofol on neural properties is manifold (Alkire et al., 2008). It affects properties of membrane ion channels, synaptic receptors and extra-synaptic receptors, see Franks and Lieb (1994) for a review. Kitamura et al. (2002) have revealed in an experimental study how propofol affects post-synaptic phasic responses of inhibitory synapses to spontaneous neurotransmitter release. They have found that the decay time constant decreases with increasing anesthetic blood concentration, the charge transfer increases while the amplitude of the responses remains constant. Some previous studies (Hutt and Longtin, 2009; Hindriks and van Putten, 2012) have implemented these effects for a bi-exponential synaptic response function. The present work considers an exponential decay phase due to its mathematical simplicity, which nevertheless reflects the major anesthetic impact. To this end, the phasic response at inhibitory synapses is determined by the decay time constant and the response amplitude. Introducing the parameter *p* = τ_2_(*p*)/τ_2_(1), *p* = 1 reflects the absence of anesthetic agents and increases of the anesthetic concentration yields an increase of *p*. In addition Kitamura et al. (2002) have shown that the amplitude in cortical neurons remains constant or changes slightly only, i.e., *h*(0) = *a*/τ_2_(*p*)≈ const and the charge transfer ρ(*p*) = *a* increase with increasing anesthetic concentration. In the case of constant the model

(9) $$\tau_{2}(p)=\tau_{2}(1)p,\;a_{i}(p)=H_{0}p.$$

with the constant *H*_0_ > 0. These choices of anesthetic actions reflect different synaptic mechanism. The first relation reflects the increase of the time constant with increasing anesthetic concentration, and the second one both the constant amplitude and the resulting increasing charge transfer. However, synapses may have different properties in different brain areas, e.g., synapses in cortico-thalamic connections increase their amplitude with increasing propofol concentrations (Ying and Goldstein, 2005). Hence the relations (Equation 9) are specific properties of inhibitory synapses on cortical neurons only.

### 3.2. The linear model

The following investigation considers the stability of stationary states and spectral properties of small deviations about them. These small deviations represent fluctuating currents on the dendritic trees of the neurons in the population generating an electric field on the scalp. They originate from fluctuations in the neuron membranes or from spontaneous neurotransmitter emission at synapses. The generated electric field is measured in terms of voltage differences between two spatial locations on the scalp which is the electroencephalogram (EEG) (Nunez and Srinivasan, 2006).

The small fluctuations about the stationary states *u*_*e*_(*x*, *t*) = *V*_*e*_(*x*, *t*) − *V*^0^_*e*_, *u*_*i*_(*x*, *t*) = *V*_*i*_(*x*, *t*) − *V*^0^_*i*_ obey

$$\begin{array}{l} \;\tau_{1}\frac{du_{e}(x,t)}{dt}=-u_{e}(x,t)+a_{e}S_{e}^{\prime }\int_{D}K_{e}(x-y)(u_{e}(y,t)-u_{i}(y,t))dy+\xi (x,t)\\ \tau_{2}(p)\frac{du_{i}(x,t)}{dt}=-u_{i}(x,t)+a_{i}(p)S_{i}^{\prime }\int_{D}K_{i}(x-y)(u_{e}(y,t)-u_{i}(y,t))dy \end{array}$$

with the somatic non-linear gain *S*′_*e*, *i*_ = *dS*_*e*, *i*_(*x*)/*dx* at *x* = {*V*^0^_*e*_, *V*^0^_*i*_}. By virtue of the finite spatial domain, the small deviations about the stationary state may be expanded into a discrete infinite Fourier series. The major contribution of neuronal activity to encephalographic activity on the scalp is modeled successfully by a spatially constant Fourier mode *u*_*e*_(*x*, *t*) = *x*(*t*), *u*_*i*_(*x*, *t*) = *y*(*t*) (Nunez and Srinivasan, 2006). Then *x*(*t*), *y*(*t*) obey

(10) $$\begin{array}{l} \;\tau_{1}\frac{dx(t)}{dt}=(-1+N_{1})x(t)-N_{1}y(t)+\gamma (t)\\ \tau_{2}(p)\frac{dy(t)}{dt}=N_{2}x(t)+(-1-N_{2})y(t) \end{array}$$

with the synaptic non-linear gains $N_{1}=a_{e}S_{e}^{\prime }{\tilde{K}}_{e}(0)\sqrt{L},\;N_{2}=N_{2}(p)=a_{i}(p)S_{i}^{\prime }{\tilde{K}}_{i}(0)\sqrt{L}$, the spatial Fourier transform of the kernels ${\tilde{K}}_{e}(k),\;{\tilde{K}}_{i}(k)$ and the spatial Fourier transform of the external noise at zero wavenumber γ(*t*). We point out that 〈γ(*t*)〉 = 0, 〈γ(*t*)γ(*T*)〉 = 2*D*δ(*t* − *T*).

### 3.3. Stability analysis

To study the dynamics about the stationary state, at first let us neglect the external input ξ(*t*) since in a first approximation the stability of the linear system does not depend on the external input. Then the stationary state is asymptotically stable if the characteristic equation of Equation (10)

(11) $$\lambda^{2}-\lambda Tr+det=0$$

with λ ∈ ℂ and

(12) $$Tr=\frac{N_{1}-1}{\tau_{1}}-\frac{N_{2}+1}{\tau_{2}}$$

(13) $$det=\frac{N_{1}N_{2}}{\tau_{1}\tau_{2}}-\frac{N_{1}-1}{\tau_{1}}\frac{N_{2}+1}{\tau_{2}}.$$

has solutions *Re*(λ) < 0. Here *Tr* and *det* are the trace and determinant of the linear matrix in Equation (10), respectively. In addition, the stationary state is a stable focus if *Im*(λ) = Ω ≠ 0, Ω ∈ ℝ and

$$\Omega^{2}=\frac{N_{1}N_{2}}{\tau_{1}\tau_{2}}-\frac{1}{4}{\left(\frac{N_{1}-1}{\tau_{1}}+\frac{N_{2}+1}{\tau_{2}}\right)}^{2}.$$

We observe immediately from Equation (12), that the stable focus is asymptotically stable if *Tr* < 0 or *N*_1_ < 1 for all other parameters and the system may lose stability only if *N*_1_ > 1. Since the present work aims to give conditions for certain oscillation frequencies in the population, the subsequent part of the work considers stable foci only. In addition the inhibitory synaptic time scale τ_2_ = τ_2_(*p*) depends on the anesthetic concentration, but not τ_1_. Thus τ_1_ is treated as a constant.

The new variables *a* = (*N*_1_ − 1)^2^, *b* = *N*_1_*N*_2_ − *N*_1_ + *N*_2_ + 1 and *c* = (*N*_2_ + 1)^2^ depend solely on *N*_1_, *N*_2_, and simplify the notation in the following analysis. Then for *N*_1_ > 1, stable foci stipulate

(14) $$Tr=\frac{\sqrt{a}}{\tau_{1}}-\frac{\sqrt{c}}{\tau_{2}}<0\text{ or }\frac{\tau_{2}}{\tau_{1}}<\sqrt{\frac{c}{a}},$$

(15) $$\Omega =\frac{1}{2}\sqrt{\frac{1}{\tau_{1}}\left(\frac{2b}{\tau_{2}}-\frac{a}{\tau_{1}}-\frac{c\tau_{1}}{\tau_{2}^{2}}\right)}\in \mathbb{R}.$$

The last equation implies that the determinant is positive definite, i.e.,

$$d(\tau_{2})=a\tau_{2}^{2}-2b\tau_{1}\tau_{2}+c\tau_{1}^{2}<0.$$

**Figure 1:**
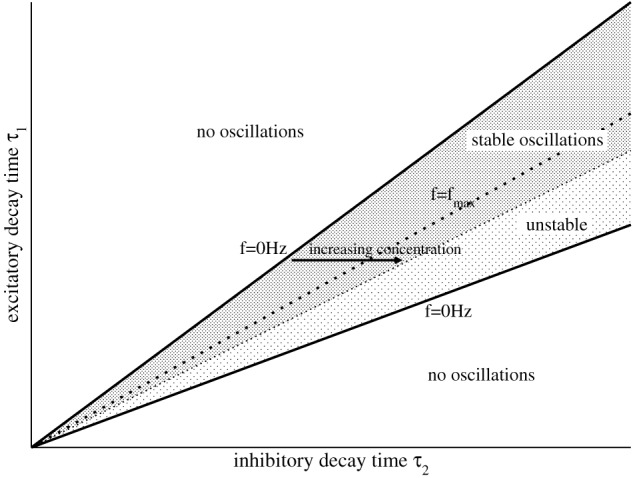
**Illustration of the parameter areas which exhibit stable, unstable, and no oscillations.** The upper and lower solid lines denote τ^−^_2_/τ_1_ and τ^+^_2_/τ_1_, respectively. The dotted line denotes the maximum frequency *f*_max_ = 2πω_*m*_ with τ_2_/τ_1_ = *b*/*c*, cf. Equation (20), and the stability threshold is given by $\tau_{2}/\tau_{1}=\sqrt{c/a}$, see Equation (14).

A comparison to Equation (15) reveals that *d*(τ_2_) = 0 leads to Ω = 0 which permits to compute the range of τ_2_ for which the system exhibits stable foci:

(16) $$\begin{array}{l} \frac{\tau_{2}^{-}}{\tau_{1}}\leq \frac{\tau_{2}}{\tau_{1}}\leq \frac{\tau_{2}^{+}}{\tau_{1}}\\ \frac{\tau_{2}^{-}}{\tau_{1}}=\frac{b}{a}\left(1+\sqrt{1-\frac{ac}{b^{2}}}\right),\;\frac{\tau_{2}^{+}}{\tau_{1}}=\frac{b}{a}\left(1-\sqrt{1-\frac{ac}{b^{2}}}\right) \end{array}$$

implying (*b*/*a*)^2^ > *c*/*a*. Condition (16) constrains the relation of both synaptic time scales τ_2_/τ_1_ by *N*_1_ and *N*_2_, see Figure 1 for the corresponding parameter space.

**Figure 2:**
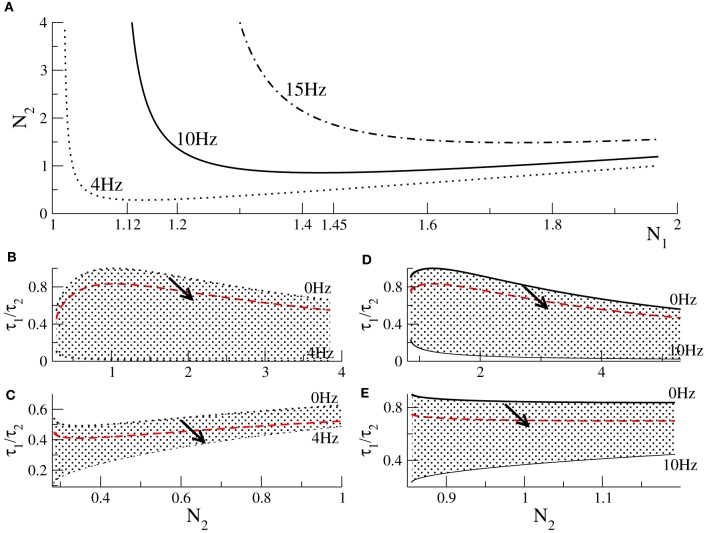
**Conditions for Hopf-instabilities and the effect of propofol action. (A)** threshold of Hopf-instability at 4, 10, and 15 Hz dependent on *N*_1_, *N*_2_. Panels **(B,C)** show parameters for Hopf-instabilities at 4 Hz dependent on *N*_2_ for which the corresponding values of *N*_1_ obey *N*_1_ < *N*_1,min_ = 1.12 and **(C)** *N*_1_ ≥ 1.12, respectively. Panels **(D,E)** show parameters for Hopf-instabilities at 10 Hz for *N*_1_ < *N*_1,min_ = 1.45 and *N*_1_ ≥ 1.45, respectively. In each panel **(B–E)** the top line reflects no oscillations, i.e., *f* = 0 Hz, and the bottom line denotes the corresponding stability threshold. Hence the shaded areas include parameters for which the system exhibits stable oscillations. The black solid and dotted lines reflect the situation of no anesthetic action, i.e., *p* = 1.0. The red-dashed line denotes the values of τ_1_/τ_2_ × *p* with *p* = 1.3. The arrows illustrate how the parameters change when anesthetic action is increased, i.e., when *p* is increased.

To learn more about the dynamics of the model, we consider parameters yielding strong oscillations with a predefined frequency, such as *f* = 4 Hz (δ-band), *f* = 10 Hz (α-band), or *f* = 15 Hz (β-band) as observed in experiments (Cimenser et al., 2011). To this end, we fix the frequency Ω = 2π*f* in Equation (15). Then inserting the threshold condition $\tau_{2}/\tau_{1}=\sqrt{c/a}$ given in Equation (14) into (15) for a fixed τ_1_ yields a relation between *N*_1_ and *N*_2_, see Figure 2A. Similarly the condition Ω = 0 given by Equation (16) determines the values of *N*_1_ and *N*_2_ at the threshold of oscillations. Figures 2B–E plot the parameter space τ_2_/τ_1_ − *N*_2_ where the system exhibits stable oscillations. The figure also shows how the values change when increasing *p* (arrows in panels), i.e., increasing the propofol concentration, and it turns out that the system always approaches the stability threshold.

### 3.4. The spectral power

To compute the power spectrum, we employ the method of Greens function. Equations (10) show that the solutions of the system obey

$$x(t)=\int_{-\infty }^{\infty }\text{​}G_{11}(t-\tau )\gamma (\tau )d\tau ,\;y(t)=\int_{-\infty }^{\infty }G_{21}(t-\tau )\gamma (\tau )d\tau$$

with the Greens functions

(17) $$G_{11}(t)=\frac{1}{2\pi }\int_{-\infty }^{\infty }\frac{i\omega +(N_{2}+1)/\tau_{2}}{P(i\omega )}e^{i\omega t}d\omega$$

(18) $$G_{21}(t)=-\frac{1}{2\pi }\int_{-\infty }^{\infty }\frac{N_{2}/\tau_{2}}{P(i\omega )}e^{i\omega t}d\omega$$

and *P*(*i*ω) = −ω^2^ − *i*ω*Tr* + *det* using the definitions of *Tr* and *det* in Equations (12) and (13). In fact, *P*(*i*ω) in Equations (17) and (18) is the characteristic polynom in Equation (11) for λ = *i*ω.

In the following in a good approximation (Nunez and Srinivasan, 2006) we assume that the experimental encephalographic data that we want to model originates from the excitatory synapses, i.e., *x*(*t*). This assumption is reasonable since the encephalographic activity is observed due to aligned apical dendritic branches and more excitatory synapses than inhibitory synapses are located on the apical branches of dendrites on cortical neurons. However, it is also possible to derive the power spectral density for the difference of excitatory and inhibitory potentials *x*(*t*) − *y*(*t*) (Hutt and Longtin, 2009). Now the application of the residue theorem allows to compute the integrals in Equation (17) by

$$G_{11}(t)=\frac{1}{2\pi i}\sum_{n=1}^{2}\text{Res}(z_{n})\Theta (t)$$

with the residues

$$\begin{array}{l} \text{Res}(z_{1})=\frac{1}{2\pi i}\frac{\lambda_{1}+(N_{2}+1)/\tau_{2}}{\lambda_{1}-\lambda_{2}}e^{\lambda_{1}t},\\ \text{Res}(z_{2})=\frac{1}{2\pi i}\frac{\lambda_{2}+(N_{2}+1)/\tau_{2}}{\lambda_{2}-\lambda_{1}}e^{\lambda_{2}t} \end{array}$$

and the roots λ_1_ = λ^*^_2_ = *R* + *i*Ω of the characteristic polynom *P*(λ) = 0 in Equation (11). This solution is valid if and only if Res(λ_*n*_) = *R* < 0. These yields

$$G_{11}(t)=e^{Rt}\left(\frac{R+Z}{\Omega }\sin(\Omega t)+\cos(\Omega t)\right)\Theta (t)$$

with *Z* = −(*N*_2_ + 1)/τ_2_, i.e., the Greens function of *x*, and hence *x*(*t*) itself oscillates with frequency Ω and is damped with the factor |*R*|. In other words, the solution *x* oscillates with the imaginary part of the root of the characteristic equation, and this frequency is already determined in the stability analysis.

Finally, the power spectral density *S*(ω) of *x* is the Fourier transform of the auto-correlation function 〈*x*(*t*)*x*(*t* − *T*)〉:

(19) $$\begin{array}{l} S(\omega )=2D\sqrt{2\pi }|{\tilde{G}}_{11}(\omega ){|}^{2}\\ \;=2D\sqrt{2\pi }\frac{Z^{2}+\omega^{2}}{{\left(R^{2}+\Omega^{2}-\omega^{2}\right)}^{2}+4R^{2}\omega^{2}} \end{array}$$

where ${\tilde{G}}_{11}(\omega )$ is the Fourier transform of the Greens function *G*_11_(*t*).

### 3.5. The frequency of maximum power spectral density

According to the reversed-engineering approach motivated in the previous section, this section aims to derive further conditions on model constants for certain oscillations close to instabilities. This vicinity to the stability threshold guarantees a small damping factor *R* and thus the power peak is located close to Ω.

There is a maximum frequency ω_*m*_ of the oscillation frequency with respect to τ_2_ given in Equation (15) and reached at

(20) $$\frac{\tau_{2}}{\tau_{1}}=\frac{b}{c}\;\to \;\omega_{m}=\frac{1}{2}\sqrt{c/\tau_{2}^{2}-a/\tau_{1}^{2}}\text{ if }\frac{\tau_{2}}{\tau_{1}}<\sqrt{c/a}.$$

The last condition is identical to the stability condition (14), i.e., stable systems always have a non-vanishing maximum frequency ω_*m*_ > 0. Together with Equation (16):

• If $b/c<\sqrt{c/a}$, then the frequency Ω may increase or decrease while increasing τ_2_ with

(21) $$\frac{d\Omega }{d\tau_{2}}>0\text{ for }\frac{\tau_{2}^{-}}{\tau_{1}}\leq \frac{\tau_{2}}{\tau_{2}}<\frac{b}{c}\;,$$

(22) $$\frac{d\Omega }{d\tau_{2}}\leq 0\text{ for }\frac{b}{c}\leq \frac{\tau_{2}}{\tau_{2}}\leq \sqrt{\frac{c}{a}}\;.$$

• If $b/c\geq \sqrt{c/a}$, then the frequency Ω increases only while increasing τ_2_ with

(23) $$\frac{d\Omega }{d\tau_{2}}>0\text{ for }\frac{\tau_{2}^{-}}{\tau_{1}}\leq \frac{\tau_{2}}{\tau_{2}}\leq \sqrt{\frac{c}{a}}\;.$$

Figure 1 shows the case $b/c<\sqrt{c/a}$. There increasing τ_2_ by increasing *p* from small frequencies on the left border for constant τ_1_ increases the oscillation frequency until reaching the dotted line, i.e., *d*Ω/*d*τ_2_ > 0. Then a further increase of *p* decreases the frequency of the oscillations again, *d*Ω/*d*τ_2_ < 0. Although this reasoning assumes that *N*_2_(*p*) does not change with *p*, it gives a first insight into the dependence of the systems oscillation frequencies on *p*.

Now considering the power spectral density (Equation 19), for *R*^4^ + *R*^2^(2Ω^2^ − 3*Z*^2^) + Ω^2^(Ω^2^ + *Z*^2^) > 0 *S*(ω) has a global maximum at

$$\Omega_{\text{peak}}=\sqrt{\Omega^{2}+R^{2}\left(1-4\frac{Z^{2}}{R^{2}+\Omega^{2}+Z^{2}}\right)}.$$

We learn that a vanishing real part of the characteristic root |*R*| yields a global maximum of the power spectral density at the imaginary part of the characteristic root Ω. From a reversed-engineer point of view, one could say that a strong peak in the experimental power spectral density reflects a characteristic root in the underlying linear system with a small real part. This way to interpret the spectral results allows to find analytical conditions for physiological parameters, as will be seen below.

If *R*^4^ + *R*^2^(2Ω^2^ −3*Z*^2^) + Ω^2^(Ω^2^ + *Z*^2^) < 0, then the system exhibits a global maximum at Ω_peak_ = 0. Since *R* and Ω depend on the anesthetic concentration, i.e., the parameter *p*, it is interesting to examine how Ω_peak_ depends on *R* and Ω:

(24) $$\frac{d\Omega_{\text{peak}}}{dR}>0\text{ for }R^{2}>Z^{2}(p)-\Omega^{2}\;\text{or}\;R^{2}<Z^{2}(p)-\Omega^{2}$$

(25) $$\begin{array}{l} \frac{d\Omega_{\text{peak}}}{d\Omega }>0\text{ for }R^{2}>\sqrt{2}|Z(p)|\Omega -\Omega^{2}+Z^{2}(p)\;\\ \text{ or }R^{2}<-\sqrt{2}|Z(p)|\Omega -\Omega^{2}+Z^{2}(p) \end{array}$$

**Figure 3:**
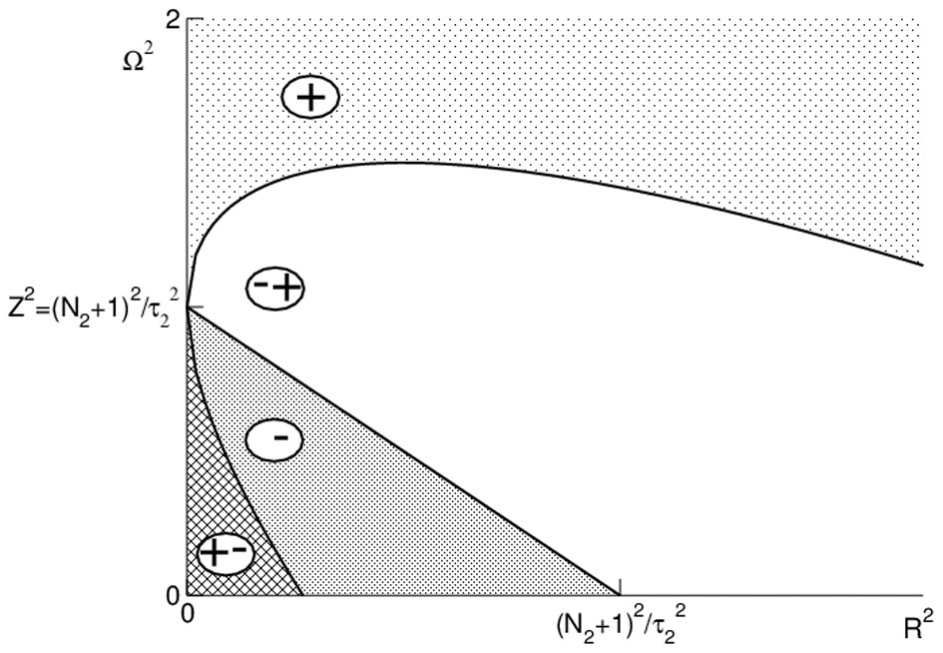
**Illustration of the conditions (24) and (25).** The regions are marked as follows: (+): *d*Ω_peak_/*d*Ω > 0, *d*Ω_peak_/*dR* > 0; (∓): *d*Ω_peak_/*d*Ω < 0, *d*Ω_peak_/*dR* > 0; (−): *d*Ω_peak_/*d*Ω < 0, *d*Ω_peak_/*dR* < 0; (±): *d*Ω_peak_/*d*Ω > 0, *d*Ω_peak_/*dR* < 0.

Figure 3 illustrates these conditions and shows that Ω_peak_ is increased or decreased subjected to values of *R* and Ω. Importantly, we observe in Figure 3 that Ω_peak_ increases with *R* and Ω for large frequencies Ω.

It remains to examine how *R*^2^ and Ω^2^ depend on *p* to finally gain the full description how Ω_peak_ changes with *p*. To this end, we consider the specific assumption (Equation 9) on the anesthetic action yielding the specific dependence of Ω^2^ and *R*^2^ to *p*

$$\begin{array}{l} \frac{d^{2}R}{dp}=-2\frac{R}{\tau_{2}(1)}\frac{1}{p^{2}}>0\\ \frac{d\Omega^{2}}{dp}=-\frac{2}{\tau_{2}(1)p^{2}}\left(A-\frac{1}{\tau_{2}(1)p}\right) \end{array}$$

with *A* = (1 − *N*_1_)/τ_1_ − *N*_2_(1)/τ_2_(1), *N*_2_(*p*) = *N*_2_(1)*p*, and τ_2_ = τ_2_(1)*p*. Defining *p*_0_ = (*N*_2_(1) + τ_2_(1)(*N*_1_ − 1)/τ_1_)^−1^ and re-calling *p* ≥ 1, then there exist two distinct cases:

• If *p*_0_ < 1, then *d*Ω^2^/*dp* > 0 for all *p*. Specifically, this stipulates

$$N_{2}(1)>1+\frac{\tau_{2}(1)}{\tau_{1}}-\frac{\tau_{2}(1)}{\tau_{1}}N_{1}.$$

• If *p*_0_ ≥ 1, then there is a small interval 1 ≤ *p* ≤ *p*_0_ for which *d*Ω^2^/*dp* ≤ 0. For larger *p*, *d*Ω/*dp* > 0. For instance, for *p*_0_ = 1.3, i.e.,

$$N_{2}(1)=1/1.3+\frac{\tau_{2}(1)}{\tau_{1}}-\frac{\tau_{2}(1)}{\tau_{1}}N_{1},$$

the frequency decreases with increasing *p* for clinically reasonable concentrations, i.e., 1 ≤ *p* ≤ 1.3 (Hutt and Longtin, 2009).

**Figure 4:**
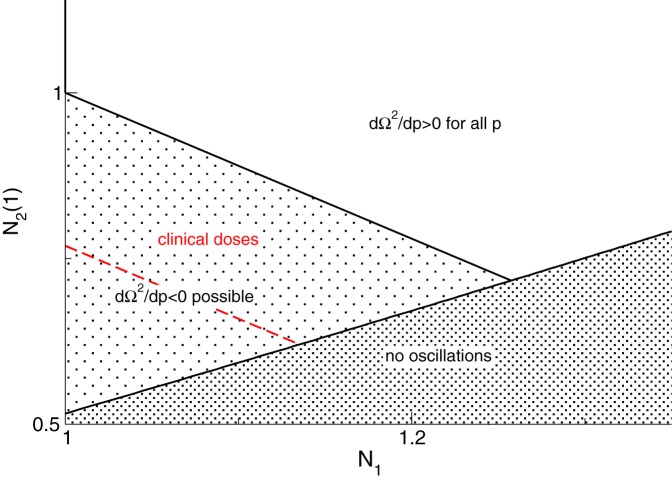
**Parameter space for different signs of *d*Ω^*2*^/*dp*.** The red line denotes *p*_0_ = 1.3 and the parameter space of clinical doses (red) implies *d*Ω^2^/*dp* < 0 for clinically reasonable drug doses, i.e., *p* ≤ 1.3. An additional parameter is *r* = τ_2_(1)/τ_1_ = 1.11.

Figure 4 illustrates the parameter space where *d*Ω^2^/*dp* has different signs and we observe that the system may exhibit either increasing or decreasing frequencies while increasing *p*.

To elucidate the systems behavior at the stability threshold, we set τ_2_(1)/τ_1_ = (*N*_2_(1) + 1)/(*N*_1_ − 1) according to Equation (14) and find *p*_0_ = 1/(2*N*_2_(1) + 1) < 1. Consequently, oscillations close to the stability threshold always increase their frequencies with increasing *p*. In contrast, for 1 < τ_2_(1)/τ_1_ « (*N*_2_(1) + 1)/(*N*_1_ − 1), i.e., systems far from the stability threshold, may exhibit values *p*_0_ > 1.

**Figure 5:**
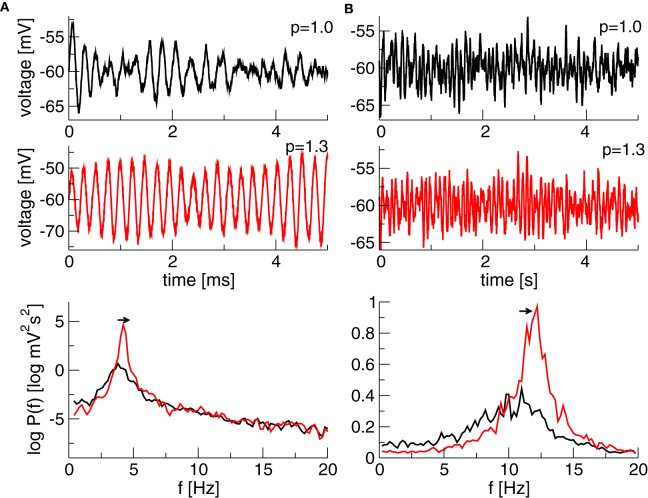
**Simulated time series of *x*(*t*) + *V*^0^_*e*_ and the corresponding power spectrum of *x*(*t*). (A)** *N*_1_ = 1.1, *N*_2_ = *p* × 0.25128, τ_2_ = τ_1_ × *p*/0.10 generating a maximum power in the δ-band. **(B)** *N*_1_ = 1.1, *N*_2_ = 0.2236 × *p*, τ_2_ = τ_1_ × *p*/0.10 generating a maximum in the α-band. Other parameters are τ_1_ = 2ms, the noise strength κ = 0.01 mV and *V*^0^_*e*_ = −60 mV. The Equation (10) is simulated with a Euler–Maryuama method for 200 s, time step was Δ*t* = 0.05 ms.

Finally, Figure 5 illustrates the temporal dynamics in two different frequency bands close to the corresponding Hopf-instabilities and shows the effect of increased propofol concentration. The power increases and the peak of maximum power moves to larger frequencies as predicted by the theory and as observed in experiments (Hindriks and van Putten, 2012).

## 4. Discussion

The introduced model considers first-order synaptic responses and take into account experimental findings on the propofol effect in synaptic GABA_*A*_-receptors in cortical neurons. It describes the evolution of neural populations on a mesoscopic level involving major properties of underlying neurons and synapses on the microscopic description level.

The analytical study reveals that the frequency of maximum power may increase or decrease with increasing anesthetic concentration subjected to the physiological constants, cf. Figure 3. In detail, close to the oscillatory instability the frequency of maximum power always increases with increasing *p* as observed in EEG (Gugino et al., 2001; Feshchenko et al., 2004; Hindriks and van Putten, 2012), whereas far from the stability threshold the maximum power frequency may also decrease as observed recently in EEG (Ching et al., 2010; Cimenser et al., 2011), cf. Figures 3, 4. The analytical treatment shows clearly that these two findings depend strongly on the physiological parameters, which are derived analytically in section 3.5, i.e., the phenomena depend on the brain area in which they are generated.

Moreover, the analytically predicted increase of the power at higher frequencies explains the power enhancement in the α- and β-band in anesthesia (Gugino et al., 2001; McCarthy et al., 2008) by a dynamic oscillatory instability. In fact, the analytical treatment in the present work suggests that power enhancement always starts from oscillatory activity at lower frequencies and are generated at slightly higher frequencies with increased concentration (Figure 5). This is in accordance to previous EEG-studies (Gugino et al., 2001; Hindriks and van Putten, 2012) showing power enhancement induced in the α- and β-band.

The analytical discussion in section 3.3 also predicts that decreasing the inhibitory time constant always moves the system toward an oscillatory instability, cf. Figure 1, and hence increases the spectral power, whereas increasing the charge transfer yields a stabilization of the system due to *dR*/*dP* < 0 and consequently a decrease of power. Hence the balance between decay prolongation and increased charge transfer decides on the change of the spectral power and the shift of the frequency peaks. Since many anesthetics share this balance in the major target GABA_*A*_-receptor (Alkire et al., 2008) and exhibit similar EEG-change (Gugino et al., 2001; Kuizenga et al., 2001), the presented work suggests that this balance reflects one of the major underlying mechanisms during the sedation phase in GA.

Previous studies (Bojak and Liley, 2005; Hindriks and van Putten, 2012) already have explained the power enhancement in anesthesia by an oscillatory instability in high-dimensional neural models. As one of the first, the present work gives analytical conditions on physiological parameters for this effect, while Bojak and Liley (2005) and Hindriks and van Putten (2012) mainly performed numerical studies. The recent work of Hindriks and van Putten (2012) resembles in some aspects the analytical approach of the present work by discussing in some detail the dynamics of superimposed oscillation modes subjected to the propofol concentration. However, no analytical conditions are given due to the higher model complexity.

It is important to point out that the current model is low-dimensional, physiologically reasonable and analytically treatable but still able to explain the neural phenomenon of the frequency shift to larger values. Bojak and Liley (2005) and Hindriks and van Putten (2012) have not performed a detailed analytical study of this phenomenon and have not derived analytical conditions under which it may occur. The current work shows that already a rather simple coupling of excitation and inhibition in cortical neural networks is sufficient to explain this phenomenon. This is concluded partially by Hindriks and van Putten (2012) based on a small numerical study, whereas the present work shows this explicitly. However, Hindriks and van Putten (2012) also argue that the cortico-thalamic feedback should be negative to gain this effect. The presented model does not need the thalamic feedback loop for the explanation.

In principle, the present work extends the work of Bojak and Liley (2005) studying just numerically a rather complicated model with tens of unknown parameters, while the current model allows to achieve some insights into the effect of few parameters. For instance, the maximum of spectral power and the corresponding frequency are highly sensitive to modification of the relation between excitatory and inhibitory synaptic time scales. This confirms the general observation that many different anesthetics share effects on the activity spectrum.

The work both supports the hypothesis of cortical generation of α-activity and predicts the presence of oscillating neural circuits where each circuit generates a certain oscillation observed experimentally. This can be observed in Figure 5 showing emerging δ- and α-activity for different parameters. Hence two neural circuits with different properties may explain the occurrence of both δ- and α-activity observed in experimental data. This interpretation of the results complements the findings of Hindriks and van Putten (2012) showing implicitly a linear decomposition into eigenmodes with corresponding eigenvalues and manifests the notion of interacting oscillation modes generated by interacting networks as observed experimentally (Fries, 2009; Spaak et al., 2012).

Of course the present model is limited since it cannot explain the increase of activity in the δ-band as observed in experiments and modeled by the previous studies. In the present work, we observe clearly that this synchronous modeling of two rhythms is not possible since the model is too low-dimensional and just can describe a single rhythm such as the α- or the δ-rhythm. Consequently further neural elements should be considered to gain this additional rhythm such as the thalamic loop. This may be possible due to the linear superposition of oscillatory activity originating from different networks. This linear superposition proposes the interaction of different sub-networks each oscillating in a certain frequency band. Future work will consider such entangled neural networks on the basis of the presented neural field to explain the spontaneous emergence or diminution of spectral peaks in experimentally observed data such as the δ-rhythms or transient phenomena such as paradoxical excitation.

### Conflict of interest statement

The author declares that the research was conducted in the absence of any commercial or financial relationships that could be construed as a potential conflict of interest.
